# Chromomycin A_5_ induces bona fide immunogenic cell death in melanoma

**DOI:** 10.3389/fimmu.2022.941757

**Published:** 2022-11-09

**Authors:** Katharine Gurgel Dias Florêncio, Evelline Araújo Edson, Keilla Santana da Silva Fernandes, João Paulo Mesquita Luiz, Francisco das Chagas Lima Pinto, Otília Deusdênia Loiola Pessoa, Fernando de Queiroz Cunha, João Agostinho Machado-Neto, Diego Veras Wilke

**Affiliations:** ^1^Drug Research and Development Center, Department of Physiology and Pharmacology, School of Medicine, Federal University of Ceara, Ceara, Brazil; ^2^Center for Research in Inflammatory Diseases (CRID), Ribeirão Preto Medical School, University of São Paulo, São Paulo, Brazil; ^3^Department of Organic and Inorganic Chemistry, Sciences Center, Federal University of Ceara, Ceara, Brazil; ^4^Department of Pharmacology, Institute of Biomedical Sciences, University of Sao Paulo, São Paulo, Brazil

**Keywords:** chromomycin, immunogenic cell death, metastatic melanoma, drug discovery, cancer immunotherapy, marine natural products, autophagy, anticancer

## Abstract

**Purpose:**

Some first-line cytotoxic chemotherapics, *e.g.* doxorubicin, paclitaxel and oxaliplatin, induce activation of the immune system through immunogenic cell death (ICD). Tumor cells undergoing ICD function as a vaccine, releasing damage-associated molecular patterns (DAMPs), which act as adjuvants, and neoantigens of the tumor are recognized as antigens. ICD induction is rare, however it yields better and long-lasting antitumor responses to chemotherapy. Advanced metastatic melanoma (AMM) is incurable for more than half of patients. The discovery of ICD inducers against AMM is an interesting drug discovery strategy with high translational potential. Here we evaluated ICD induction of four highly cytotoxic chromomycins A (CA_5-8_).

**Methods:**

ICD features and DAMPs were evaluated using several *in vitro* techniques with metastatic melanoma cell line (B16-F10) exposed to chromomcins A_5-8_ such as flow cytometry, western blot, RT-PCR and luminescence. Additionally *in vivo* vaccination assays with CA_5_-treated cells in a syngeneic murine model (C57Bl/6) were performed to confirm ICD evaluating the immune cells activation and their antitumor activity.

**Results:**

B16-F10 treated with CA_5-8_ and doxorubicin exhibited ICD features such as autophagy and apoptosis, externalization of calreticulin, and releasing of HMGB1. However, CA_5_-treated cells had the best profile, also inducing ATP release, ERp57 externalization, phosphorylation of eIF2α and altering expression of transcription of genes related to autophagy, endoplasmic reticulum stress, and apoptosis. Bona fide ICD induction by CA_5_ was confirmed by vaccination of C57BL/6 mice with CA_5_-treated cells which activated antigen-presenting cells and T lymphocytes and stimulated antitumor activity.

**Conclusion:**

CA_5_ induces bona fide immunogenic cell death on melanoma.

## 1 Introduction

Advanced metastatic melanoma (AMM) is the most aggressive skin cancer, and is a serious concern due to increasing incidence in recent decades. Chemotherapy with dacarbazine and temozolomide was the standard of care in metastatic melanoma until 2011, however with no benefit for overall survival (1). Immunological therapy with IL-2 induces long-lasting responses in a small subset of patients, albeit with a high rate of severe toxicities (1, 2). Advances in AMM treatment have yielded better agents based on target therapies, such as BRAF and MEK inhibitors (3) and immunological checkpoint inhibitors, e.g. anti-PD-L1, anti-PD-1 and anti-CTLA-4 antibodies (1, 4). Despite these advances, more than half of patients still do not experience a satisfactory clinical response. Thus translational research should pay particular attention to the non-responders subset of patients, aiming to shift immune-cold tumors into immune-hot ones (1).

The immunogenic effect of first-line chemotherapy and some radiotherapy treatments have been revealed as the hidden ally which improves responses of patients (5–7). Treatment with inducers of immunogenic cell death (ICD) produces a specific antitumor immunity, which potentiates therapeutic efficacy (7–9). ICD is a rare type of regulated cell death characterized by the activation of the adaptive immune system in the presence of cell death antigens, especially from cancer cells. Only 5% of chemotherapeutics the arsenal approved by the Food and Drug Administration of USA for cancer treatment are validated ICD inducers. However, they are first-line agents in the clinic and among the most used in the world including anthracyclines, taxanes and oxaliplatin (10). This cell demise occurs under strong cellular stress, including autophagy and endoplasmic reticulum (ER) stress, and release of a constellation of damage-associated molecular patterns (DAMPs), the signals required to recruit and activate immune cells, such as antigen-presenting cells (APCs) and lymphocytes. The most important DAMPs in ICD include ATP, high mobility box group-1 (HMBG1), a nuclear non-histone nuclear factor, chaperones, specially calreticulin, and heat-shock proteins 70 and 90, annexin A1, CXCL10 and type I interferon (6, 11). In addition to *in vitro* evaluation of cell demise associated with cell stress and release of DAMPs, a vaccination assay is required to validate induction of ICD through an anti-tumor response *in vivo* (5, 12). Clinical evidence also supports ICD as a sensitizer to PD-1/PD-L1 blockade (13). Thus identification of ICD inducers against AMM is a promising strategy for early identification of anticancer candidates with high expectation of translational success.

Chromomycins and mithramycins are promising antitumor antibiotic aureolic acids (14). In 1970, mithramycin was approved for use in testicular cancer (15), and chronic and acute myeloid leukemia (16, 17). Recent studies have shown mithramycin inhibition of drug-resistant cancer-initiating stem cells, important players in the disease relapse (14). Mithramycins also have been reported as an inhibitor of P-glycoprotein, a transmembrane efflux pump related to resistance to multiple drugs in cancer cells (18).

Chromomycin A_3_ has antitumor activity, reversibly binding to minor DNA grooves by interacting with cytosine and guanine (CG)-rich DNA regions in the presence of Mg^2+^, preventing replication and transcription (19, 20). Guimarães et al. (21) showed chromomycin A_2_ induces autophagy in metastatic melanoma cells, MALME-3M. The pre-apoptotic autophagy is related to the immunogenic outcome of cancer cell death (22). Recently we obtained four cytotoxic dextrorotatory chromomycins A (CA_5_, CA_6_, CA_7_, and CA_8_) from the actinobacteria *Streptomyces* sp. BRA-384 which displayed IC_50_ values against five tumor cells from the pM to nM range (23). CA_5_ binds to the transcription factor T-box 2 (TBX2), which also may be related to its antiproliferative effect and antimetastatic potential (24). Because CA_5-8_ are highly cytotoxic (23) and likely to induce autophagy of melanoma cells (21), we hypothesized these compounds were bona fide ICD inducers. Here we investigated the induction of ICD by CA_5-8_ on an AMM model, which could provide additional evidences of chromomycins as promising anticancer agents.

## 2 Materials and methods

### 2.1 Reagents

Chromomycins (CA_5_, CA_6_, CA_7_, and CA_8_) were obtained as previously described by Pinto et al., 2019 (23). Doxorubicin and dimethyl sulfoxide (DMSO) were purchased from Sigma-Aldrich (Missouri, USA). All cytotoxic compounds were diluted in DMSO.

### 2.2 Cell culture

The murine metastatic melanoma B16-F10 cell line was purchased from Banco de células do Rio de Janeiro (Rio de Janeiro, Brazil) and cultured following the manufacturer’s instructions.

### 2.3 Animals

We used C57BL/6 mice (female, 18–20 g) 6-8 weeks-old, free of ecto and endoparasites, obtained from the animal houses of the Federal University of Ceara, Brazil and University of Sao Paulo, Brazil. Animals were housed in cages under a 12:12 h light-dark cycle (lights on at 6:00 a.m.) and food and water *ad libidum*. All animal handling procedures were performed following the Brazilian legislation for the use and care of laboratory animals (No 11.724/20080) after approval by Animal Ethics Committee of the Federal University of Ceara (No 3000310818) and Animal Welfare Committee of the Ribeirão Preto Medical School, University of São Paulo (No 226/2018). A total of 36 mice from the Federal University of Ceara and 31 mice from the University of São Paulo were used.

### 2.4 Antiproliferative assay

**Figure 1:**
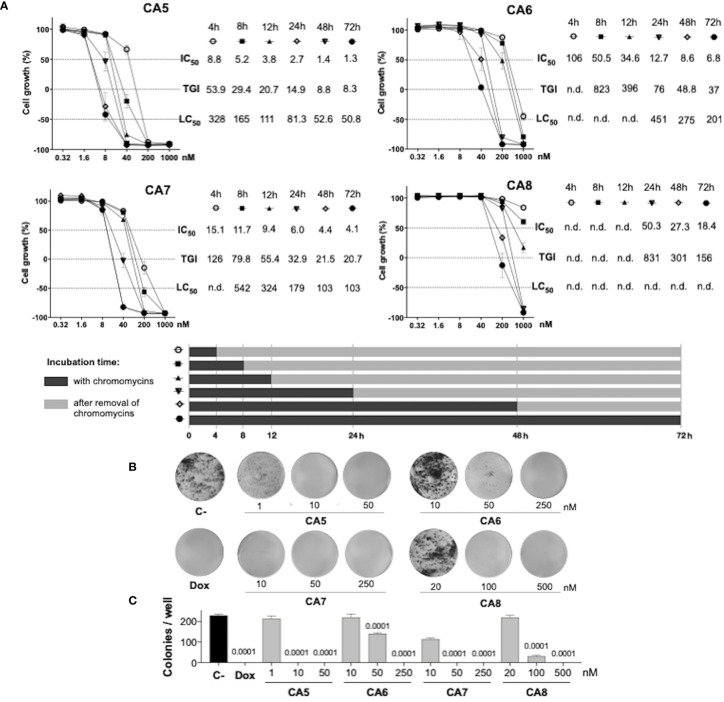
Antiproliferative profile of chromomycins A_5-8_ on metastatic melanoma B16-F10 cell line over time. **(A)**, Graphs showing the antiproliferative activity of CA_5-8_ after different exposure periods by the SRB assay. Inhibition concentration mean (IC_50_), total growth inhibition (TGI), and lethal concentration mean (LC_50_) values in nanomolar (nM) were obtained from interpolation of non-linear regression of normalized absorbance of 3 experiments performed in triplicate. **(B)**, Representative photos of the clonogenic assay, and **(C)**, Number of colonies represented as the mean ± standard deviation. 0.05% DMSO (C-) and 0.6 µM doxorubicin (Dox) were considered as a negative control and positive control, respectively. Statistical differences of treated groups versus C- are expressed as p values indicated above the columns of the groups.

Sulforhodamine B (SRB) assay was performed as described by Skehan et al., 1990 (25). CA_5-8_ 0.32 to 1000 nM (CA5, CA6, CA7 and CA8 respectively), doxorubicin at 0.6 µM (Dox) as positive control and DMSO (0.05%) as negative control were added to cells during 4 h, 8 h, 12 h, 24 h, 48 h and 72 h and antiproliferative effect evaluated after 72 h. When the exposure time was < 72h, cells were washed and replaced by fresh media (see **Figure 1**). Inhibition concentration mean (IC_50_), total growth inhibition (TGI), and lethal concentration mean (LC_50_) values were calculated from cell growth percentage normalized data through interpolation of nonlinear regression using GraphPad Prism v6 (GraphPad Software, Inc., San Diego, CA, USA).

### 2.5 Clonogenic assay

The colony-forming assay was performed according to Franken et al., 2006 (26). Briefly, cells were seeded in 6-well plates at a density of 500 cells/well and exposed to CA_5-8_ (CA5, CA6, CA7, and CA8), doxorubicin (Dox) or 0.05% DMSO (C-) for 24 hours. After this period, the medium with cytotoxic agents or DMSO was replaced by a fresh medium, and plates were analyzed daily until the DMSO control reached a high density of individualized colonies (approximately 7 days). Cells were then washed with PBS and stained with violet crystal dye (0.5% crystal violet in methanol 50% and distilled water). Total individual colonies/well were counted under a stereoscopic microscope.

### 2.6 Cell treatment for immunogenic cell death investigation

Usually, 2.5 x 10^4^ cells/mL were seeded in 24-well plates for flow cytometry assays, 96-well for ATP assay, or in Petri dish plates (90 x 15 mm) for western blot and qPCR assays, and incubated for 24 h before the treatment. Then cells were exposed to CA_5_ at 0.1µM (CA5), CA_6_ at 0.25 µM (CA6), CA_7_ at 0.25 µM (CA7) and CA_8_ at 0.5 µM (CA8), doxorubicin at 0.6 µM (Dox), as the ICD inducer positive control, and 0.05% DMSO (C-), as the negative control, and incubated for 2 hours. After treatment, cells were collected and washed with PBS before all protocols described below.

### 2.7 Flow cytometry

All flow cytometry assays were set to acquire 10,000 events excluding debris and doublets. Tumor cell stress and cell death assays were performed using a FACSVerse™ flow cytometer (BD Biosciences, San Diego, CA, USA), immunophenotyping assays were performed using a FACSCanto™ flow cytometer (BD Biosciences, San Diego, CA, USA) and splenocytes cytotoxicity assay was performed using an Attune (Thermo Fisher Scientific, USA). Data analyses were performed using FlowJo v10.6 software (Ashland, OR: Becton, Dickinson and Company). Cell suspensions were incubated with 2 µg/mL 4′,6-diamidine-2′-phenylindole dihydrochloride (DAPI, Sigma-Aldrich, Missouri, USA) for 10 minutes as the final step in all flow cytometry cell stress and cell death assays to distinguish membrane integrity and disruption, except for the acridine orange stain.

### 2.8 Acidic vesicular organelles staining with acridine orange – flow cytometry

Differential acridine orange (AO) staining is an assay that is strongly correlated with autolysosomes formation in the late step of autophagy. The AO assay was performed as described previously (27). Briefly, cells were washed with PBS and stained with 1 µg/mL AO (Sigma-Aldrich, Missouri, USA) for 30 min in the dark at room temperature. Excitation of AO-stained cells with a 488 nm laser induces green fluorescence in whole cells and red fluorescence is produced in acidic vesicular organelles (AVOs) due to AO metachromasia. AVOs were gated in a region with an increased ratio of red fluorescence. The increase of cell granularity, although nonspecific, is a relevant cell stress alteration found in autophagy and ER stress. High cell granularity was gated in the high side scatter (SSC) region.

### 2.9 Calreticulin externalization – flow cytometry

Cells were fixed with 0.25% paraformaldehyde in ice-cold PBS for 5 min and incubated with anti-CRT (Calreticulin (D3E6) XP Rabbit mAb, Cell Signaling Technology, Danvers, MA, USA, #12238) 1:300 for 40 minutes, in the dark at 4 °C. Cells were washed with a FACS buffer (FACS solution supplemented with 4% fetal calf serum) and incubated with anti-rabbit secondary antibody Alexa Fluor 488™ (#4412, Cell Signaling Technology, Danvers, MA, USA)(1:800) for 40 minutes, in the dark at 4 °C. Cells were centrifuged and resuspended in the FACS buffer for acquisition in the flow cytometer. The percentage of Ecto-CRT was counted as Low FSC/CRT^+^ population in the DAPI^-^ cells.

### 2.10 ERp57 externalization measurement – flow cytometry

Cells were fixed with 0.25% paraformaldehyde in ice-cold PBS for 5 min and incubated with anti-ERp57 (#A484, Rabbit mAb, Cell Signaling Technology, Danvers, MA, USA) 1:100 for 40 minutes, in the dark at 4 °C. Cells were washed with a FACS buffer (FACS solution supplemented with 4% fetal calf serum) and incubated with anti-rabbit secondary antibody conjugated with Alexa Fluor 488™ (1:400) for 40 minutes, in the dark at 4 °C. Cells were centrifuged and resuspended in the FACS buffer for acquisition in the flow cytometer. The mean fluorescence intensity (MFI) related to Ecto-ERp57 was evaluated in the DAPI^-^ population.

### 2.11 Evaluation of nuclear HMGB1 - flow cytometry

Cell membranes were permeabilized with 0.1% Triton X-100 solution for 5 minutes. Then cells were washed with PBS and incubated with anti-HMGB1 conjugated with phycoerythrin (PE) (# 651403, PE anti-HMGB1; Biolegend, San Diego, CA, USA) for 40 min, in the dark at 4 °C. After incubation, cells were washed, resuspended in the FACS buffer, and acquired in the flow cytometer. The HMGB1 release was estimated indirectly by the MFI decreasing in cells exposed to doxorubicin and CA_5-8_ (28).

### 2.12 Evaluation of eIF2α and P-eIF2α – flow cytometry

Cell membranes were permeabilized with 0.1% Triton X-100 solution for 5 minutes. Then cells were washed with PBS and incubated with anti-eI2Fα (#9722, Cell Signaling Technology, Danvers, MA, USA) or anti-P-eIF2α (phospho S51) (#9721, Cell Signaling Technology, Danvers, MA, USA). Antibodies (1:100) were incubated for 40 min at 4 °C. Subsequently, these cells were washed and incubated with the secondary antibody conjugated with Alexa Fluor 488 (Biolegend, San Diego, CA, USA) (1:400) for 40 minutes, in the dark at 4 °C. After cell wash and resuspension in the FACS buffer, the data were acquired by flow cytometry. MFI of eIF2α and P-eIF2α was measured in the DAPI^+^ region.

### 2.13 ATP release assay

ATP determination kit (#A22066, ThermoFisher Scientific, Inchinnan, UK) based on luciferin-luciferase conversion was carried out according to the manufacturer’s protocol. Briefly, the supernatant was centrifuged at 1200 rpm for 5 min and 10 μL of cleared supernatants of each condition were transferred to a 96-well plate for luminescence. Then, 90 μL of ATP mix reagent was added to each well. After incubation for 1 min at room temperature, we analyzed luminescence emission in the multimode microplate reader Cytation 3 (Biotek, Vermont, USA).

### 2.14 Western blot analysis

Total protein extraction was performed using a buffer containing 100 mM Tris (pH 7.6), 1% Triton X-100, 150 mM NaCl, 2 mM PMSF, 10 mM Na_3_VO_4_, 100 mM NaF, 10 mM Na_4_P_2_O_7_, and 4 mM EDTA. Equal amounts of protein were used from total extracts followed by SDS-PAGE, and Western blot analysis with the antibodies indicated, as previously described (29) Antibodies against total and cleaved PARP1 (#9542), LC3BI/II (#2775), antibody against γ-H2AX (#9718), anti-rabbit HPR (#7074), procaspase 3 (#9665), cleaved-caspase 3 (#9665) and α-tubulin (#2144) were obtained from Cell Signaling Technology (Danvers, MA, USA). Antibody binding was revealed using a SuperSignalTM West Dura Extended Duration substrate system (Thermo Fisher Scientific) and a G: BOX Chemi XX6 gel document system (Syngene, Cambridge, UK). All the experiments were repeated at least 3 times.

### 2.15 Quantitative RT-PCR

B16-F10 cells were seeded on cell culture dishes (90 x 15 mm) and treated with 0.05% DMSO, chromomycin A_5_ (0.1 µM), or doxorubicin (0.6 μM) for 24 h. Total RNA was obtained using TRIzol reagent (Thermo Fisher Scientific). cDNA was synthesized from 1 µg of RNA using a High-Capacity cDNA Reverse Transcription Kit (Thermo Fisher Scientific). Quantitative PCR (qPCR) was performed using a QuantStudio 3 Real-Time PCR System in conjunction with a SybrGreen System (Thermo Fisher Scientific) in conjunction with a SybrGreen System for the expression of *Atf4*, *Atf6*, *Atg5, Atg7, Bak1, Bad, Bax, Bcl2, Becn1, Carl, Hspa4, Hspa5 and Sqstm1* genes. *Actb* and *Hprt1* were used as reference genes. A negative ‘No Template Control’ was included for each primer pair. All procedures were performed according to the manufacturer’s instructions. Relative quantification values were calculated using the 2^-ΔΔCT^ equation (30). The heatmap was constructed using the multiple experiment viewer (MeV) 4.9.0 software (31). The network analysis was performed using modulated genes by Dox or CA5 groups using the GeneMANIA tool (32).

### 2.16 Splenocytes activation assay

Melanoma B16-F10 cells (2 x 10^5^) were seeded in 200 µL in a 96-well culture microplate and treated with saline solution (C-), as negative control, 0.6 µM doxorubicin (Dox), as positive control, and 0.1 µM CA_5_ (CA5) for 24 h prior to incubation with splenocytes. The cell culture was centrifuged for 8 min at 450 x g and washed with warm PBS. Splenocytes (2 x 10^5^ cells) were added to each well of treated melanoma cells and incubated for 2 days (33). Supernatants were harvested and stored at -20 °C for cytokine quantification. Quantification of TNF-α in the supernatant was performed by Enzyme-linked immunosorbent assay (ELISA) (R&D Systems), according to the manufacturer’s instructions.

### 2.17 Immunological effects of mice vaccinated with cells exposed to CA5

#### 2.17.1 Vaccination protocol

Vaccination assays were performed with a syngeneic mouse model as described by Gomez-Cadena et al. (28) with modifications in order to evaluate the activation of immune cells described in the sections below. A syngeneic mouse model (e.g., B16-F10 cell lines), is an appropriate approach to study cancer therapy with a functional immune system. The vaccination was performed on days -3 or -7 when the mice of the 3 experimental groups received subcutaneously into the right axilla 200 µL of 0.9% saline solution (C-), as a negative control, or 200 µL of dying B16-F10 cells pre-exposed 24 h to 0.1 µM CA_5_ (CA5) and 0.6 µM doxorubicin (Dox), as a positive control. The dying cells were obtained after exposure to cytotoxic agents for 24 h, then harvested, washed with PBS twice, and resuspended in PBS with 1.8 x 10^5^ cells/200 µL. No additional adjuvants were added. The splenocytes were obtained by maceration of spleens in a cell strainer (40 µm) using 5 mL of PBS to elute the cells.

#### 2.17.2 Immunophenotyping of dendritic cells and T lymphocytes – flow cytometry

Animals were vaccinated as described above. Three or seven days later, splenocytes from mice of C-, CA5, and Dox groups (N=5 animals/group) were obtained to evaluate activation of dendritic cells (DCs). 2 x 10^6^ cells/mL in a 96-well plate were labeled using the conjugated antibodies against CD11b PE-Cy7 (Cat # 552850, BD Bioscience), CD11c BV421 (Cat # 562782, BD Bioscience), CD45 BV510 (Cat # 563891, BD Bioscience), CD80 PE (Cat # 104708, BioLegend), CD86 APC (Cat # 17-086282, eBioscience), MHC II FITC (Cat # 553623, BD Bioscience) (34).

After seven days of vaccination, splenocytes from mice of C-, CA5, and Dox groups (N=5 animals/group) were obtained to evaluate the activation of T lymphocytes. 2 x 10^6^ cells/mL in a 96-well plate were stimulated with 50 ng/mL phorbol myristate acetate (PMA) and 500 ng/mL ionomycin for 4 hours at 37°C. Cytokine secretion was blocked with Stop Golgi (BD Bioscience). The activation profile was investigated the conjugated antibodies CD3 PE (Cat # 48-0031-82, Invitrogen), CD4 FITC (Cat # 553047, BD Bioscience), CD8 APC-Cy7 (Cat # 557654, BD Bioscience), CD69 PE (Cat # 553237, BD Bioscience), CD25 FITC (Cat # 564424, BD Bioscience) and CD44 APC (Cat # 103012, BioLegend) (35–37). Results were expressed as percentages, by the total number of cells or by the expression of the mean fluorescence intensity (MFI) detected. Data were acquired by flow cytometry.

#### 2.17.3 Cytotoxicity of splenocytes against tumor cells – flow cytometry

After seven days of vaccination, splenocytes from mice of C-, CA5, and Dox groups (N=5 animals/group) were obtained to evaluate cytotoxicity against B16-F10 cells *in vitro*. Herein cells were incubated for 10 minutes at room temperature in a hemolysis buffer containing a 9:1 ratio of 0.16 M NH4Cl and 0.01 M Tris-HCl. Cells were washed 3 times with sterile saline solution and plated at a concentration of 1 x 10^6^/mL per well in 24-well plates. After 24 h splenocytes were restimulated with saline solution (C-), as negative control, or B16-F10 cells pre-exposed to 0.6 µM doxorrubicin (Dox) and 0.1 µM CA_5_ for 24 h and incubated for additional 72 h and 120 h. After incubation, splenocytes were incubated for 5 h with viable B16-F10 cells previously labeled with 1 µM rhodamine 123 (Rh123) at effector: target ratios of 25:1, 50:1 and 100:1. After incubation cells were labeled with 5 µg/mL propidium iodide (PI) for 10 minutes and 10,000 events were acquired by flow cytometry. Dead B16-F10 cells were gated on Rh123^+^PI^+^ region, after the exclusion of debris and doublets of the analysis.

#### 2.17.4 Antitumor effect on mice

Seven days after vaccination, mice of C-, CA5, and Dox groups (N=7 animals/group) were challenged with an injection of 1.0 × 10^5^ viable B16-F10 cells in 200 μL of PBS into the left axilla subcutaneously. The size of the tumors was measured at days 12, 15, and 17 with digital calipers. The tumor volume was calculated using the following formula: tumor volume (in mm^3^) = [(width)^2^ × length]/2.

### 2.18 Immunogenic cell death index

In order to compare the putative ICD potential among compounds used in our pipeline, we scored a total of eight parameters related to cell stress (LC3BII and AVOs), cell death (caspase 3 activated, cleaved PARP1 and membrane disruption), and DAMPs (CRT externalized, releasing of HMGB1 and ATP) from assays performed with CA_5-8_ and doxorubicin as well (SI.2). The significant differences (*p < *0.05) of treated groups compared to C- were scored = 1 for cell stress and cell death parameters and = 2 for DAMP detection. Groups with p > 0.05 compared to C- were scored = 0. The sum of scores for each compound was considered an ICD index, which aided us to understand the overall immunogenic potential profile of compounds tested on tumor cells *in vitro* in this study.

### 2.19 Statistical analysis

All statistics were performed in GraphPad Prism v6 (GraphPad Software, LLC, San Diego, CA, USA). Shapiro-Wilk was used to test the normal distribution of results. Data are expressed as means ± standard deviation of the mean (SD). Comparisons between C- and treated groups were performed using the One-Way Analysis of Variance (ANOVA) followed by Dunnet´s post-test for parametric data and using Kruskal-Wallis followed by Dunn’s post-test for nonparametric data. A *p < *0.05 value was considered significant.

## 3 Results

### 3.1 Chromomycins A_5-8_ are highly cytotoxic at multiple time exposures

Initially, we performed concentration-effect curves with CA_5-8_ varying time exposure to determine their antiproliferative profile against metastatic melanoma B16-F10 cells (**Figure 1**). CA_5_ and CA_7_ were the most potent compounds, depicting low to mid nM cytostatic and cytotoxic effects respectively even at short time exposures (**Figure 1A**). In addition, CA_6_ also displayed a similar profile at longer exposures. CA_8_ induced a potent cytostatic effect, however, it failed to show cytotoxicity at the nM range. Additionally, CA_5-8_ inhibited colony formation at the nM range of B16-F10 cells incubated for 24 h (**Figures 1B, C**).

### 3.2 Chromomycins A_5-8_ induce apoptosis

**Figure 2:**
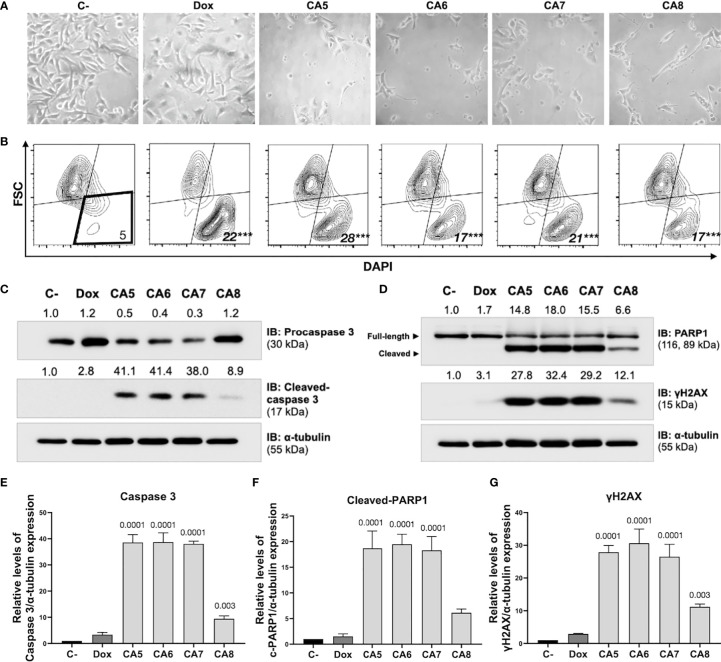
Chromomycins A_5-8_ induce cellular morphological changes and cell death. B16-F10 melanoma cells were treated with DMSO (C-), as a negative control, doxorubicin (Dox), as a positive control, and CA_5-8_ (CA5-8) for 24 h. **(A)** Phase contrast photomicrographs (200x). **(B)** Representative contour plot graphs of the cell death subpopulation gated in the low forward scatter (FSC) and DAPI^+^ region by flow cytometry. **(C)** Expression of activated caspase 3 obtained by Western blot. **(D)** Expression of cleaved PARP1 and γH2AX obtained by Western blot. Values associated with test proteins were normalized to standard α-tubulin for the relative expression measure. **(E-G)**, column graphs of Western blot analysis. Data presented as mean of 3 independent experiments. Flow cytometry analysis was performed in triplicate. ***p value = 0.001 of treated groups compared to the C-.

Immunogenic cell death (ICD) determination requires confirmation of early and late apoptotic features (12). B16-F10 cells depicted typical apoptosis features after exposure to CA_5-8_ such as cell shrinkage (**Figures 2A, B**), caspase 3 activation (**Figures 2C, E**) and PARP1 cleavage (**Figures 2D, F**). Notably, CA_5-7_ induced intense caspase 3 activation and PARP1 cleavage compared to the CA_8_, while the Dox group was similar to C-. All treated groups increased (p < 0.0001) the cell death population subset (**Figure 2B**). Similar to apoptotic markers, CA_5-8_ also induced an increase of γ-H2AX levels, and Dox elicited a mild increase of this marker of DNA damage (**Figures 2D, G**).

### 3.3 Chromomycins A_5-8_ induce autophagy

**Figure 3:**
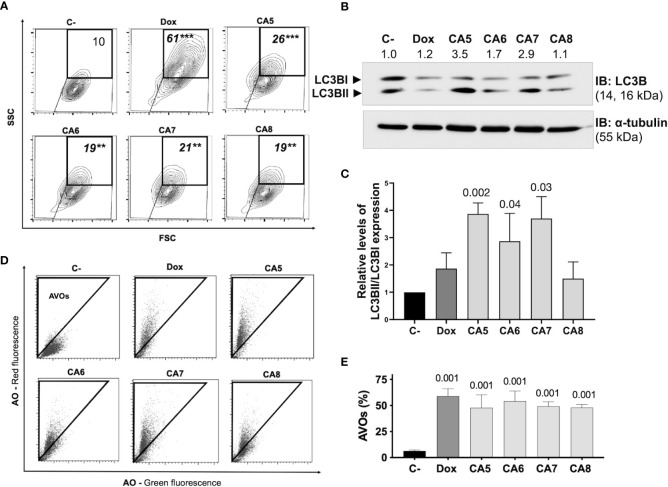
Chromomycins A_5-8_ induce autophagy. B16-F10 melanoma cells were treated with DMSO (C-), as a negative control, doxorubicin (Dox), as a positive control, and CA_5-8_ (CA5-8) for 24 h. **(A)** Representative contour plot graphs of cells with granularity gated on high side scatter (SSC) and average forward scatter (FSC) region by flow cytometry and **(B)** Expression of LC3BII and LC3BI obtained by Western blot and **(C)**, column graph of Western blot analysis. **(D)**, Representative dot plots of acridine orange (AO) staining. Acidic vesicular organelles (AVOs) were gated on the region with increased red fluorescence by flow cytometry and **(E)** the graph showing the percentage of AVOs. Data presented in graphs as mean ± standard deviation of 3 independent experiments. The AVOs detection was performed in triplicate. The *p* values of C- compared to the treated groups are above each treated group. **p=0.01 and ***p=0.001.

ICD inducers elicit cell stress associated with cell demise. Autophagy and endoplasmic reticulum (ER) stress are phenotypic changes observed quite often with ICD (38). CA_5-8_ and Dox significantly increased the granularity of B16-F10 cells (**Figure 3A**). Additionally, CA_5-8_ and Dox increased LC3BII expression (**Figures 3B, C**) and cells with acidic vesicular organelles (AVOs) (**Figures 3D, E**). These data suggest the autophagy induction by chromomycins on B16-F10 cells. Nevertheless, the relative levels of LC3BII and caspase 3 activated expression suggest a stronger apoptosis induction than autophagy on Dox and CA_5-8_ (SI.1). This is suitable, once autophagy contributes to ICD and cell death resistance as well.

### 3.4 Chromomycins A_5-8_ induce the release of ICD related DAMPs

**Figure 4:**
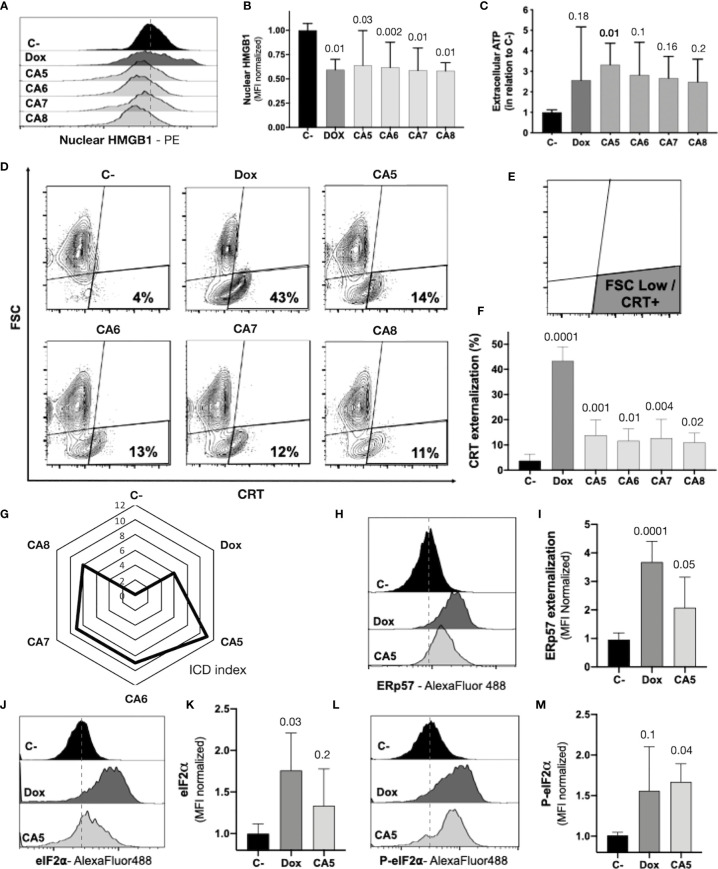
Chromomycins A_5-8_ induce the release of immunogenic cell death DAMPs. B16-F10 melanoma cells were treated with DMSO (C-), as a negative control, doxorubicin (Dox), as a positive control, and CA_5-8_ (CA5-8) for 24 h. **(A)** Representative histograms of nuclear HMGB1 evaluated by flow cytometry and **(B)** graph depicting normalized median fluorescence intensity (MFI) of nuclear HMGB1. **(C)** Normalized extracellular ATP levels measured by luminescence. **(D)** Representative contour plot graphs of calreticulin (CRT) vs cell size (forward scatter, FSC) by flow cytometry, **(E)** Illustration of the gated region of CRT+ cells with low FSC and **(F)** graph depicting the percentage of CRT+ cells. **(G)** Radar graph of the index of immunogenic cell death (ICD index). Details of ICD index are described in SI.2. **(H)** Representative histograms of ERp57 evaluated by flow cytometry and **(I)** graph depicting normalized MFI of ERp57. **(J)** Representative histograms of eIF2α evaluated by flow cytometry and **(K)**, graph depicting normalized MFI of eIF2a. **(L)** Representative histograms of cells with eIF2α phosphorylated at serine 51 (P-eIF2α) evaluated by flow cytometry and **(M)** graph depicting normalized MFI of P-eIF2a. Data presented in graphs as mean ± standard deviation of 3 independent experiments performed in triplicate. The *p* values of C- compared to the treated groups are above each treated group.

The regulated cell death of B16-F10 exposed to CA_5-8_ associated with cell stress is a minimal requirement observed in ICD (**Figures 2**, **3**). However, the immunogenicity of ICD depends on the damage-associated molecular patterns (DAMPs) releasing. Several DAMPs related to ICD have been reported so far. Despite this, only a few examples are highly recurrent in ICD, such as secretion of ATP and HMBG1, a nuclear non-histone nuclear factor, and externalization to the plasma membrane of CRT, a lumenal chaperone of the ER (6, 11, 12). We observed nuclear HMGB1 decreasing in B16-F10 cells incubated with CA_5-8_ and Dox (**Figures 4A, B**). ATP levels increased significantly, compared to C-, in supernatants of CA_5_ exposed cells, while Dox and CA_6-7_ did not change the level of this DAMP (**Figure 4C**). Additionally, CA_5-8_ induced CRT externalization in the shrunken cells subpopulation (**Figures 4D–F**) and Dox as well. ATP and HMGB1 act as classic DAMPs, with chemoattractant and activation roles on APCs as ligands of purinergic receptors (P2Y2 and P2X7) and tool-like receptor 4 respectively. The ecto-CRT is a phagocytic signal recognized by CD91 that promotes antigen presentation of tumor neoantigens in presence of HMGB1 activation (12, 39).

ICD is a complex phenomenon in which multiple phenotypic changes are required to allow the proper immune system activation. In order to compare the putative ICD potential among compounds used in our pipeline, we scored a total of eight parameters related to cell stress, cell death, and DAMPs from assays performed with CA_5-8_ and Dox as well (SI.2). The sum of scores for each compound was considered an ICD index, which aided us to understand the overall immunogenic potential profile of compounds tested in this study. CA_5_ depicted the highest ICD index followed by CA_6_ and CA_7_, CA_8_ and Dox respectively (**Figure 4G**). From this point, we focused on further analyses of the effects of CA_5_.

The co-externalization of ERp57 with CRT is the actual “eat me” signal for phagocytosis by the APCs with an activation outcome. The B16-F10 cells exposed to CA_5_ and Dox presented ERp57 externalization (**Figures 4H, I**). ER stress is an important ICD driver related to externalization of CRT and ERp57, and could initiate autophagy and apoptosis as well. The eIF2α is an ER stress protein involved in ICD. Dox-treated cells induced an increase of eI2Fα (**Figures 4J, K**), while CA_5_ treatment did not change the level of this protein. Nevertheless, the phosphorylation of eI2Fα at Ser51 is the crucial ICD signaling (40, 41). Cells incubated with CA_5_ elicited a significant activation of this protein (**Figures 4L, M**). Curiously, Dox did not increase P-eIF2a levels despite the increase of the overall levels of eIF2a.

### 3.5 CA_5_ impacts gene expression related to autophagy, ER stress, and apoptosis

**Figure 5:**
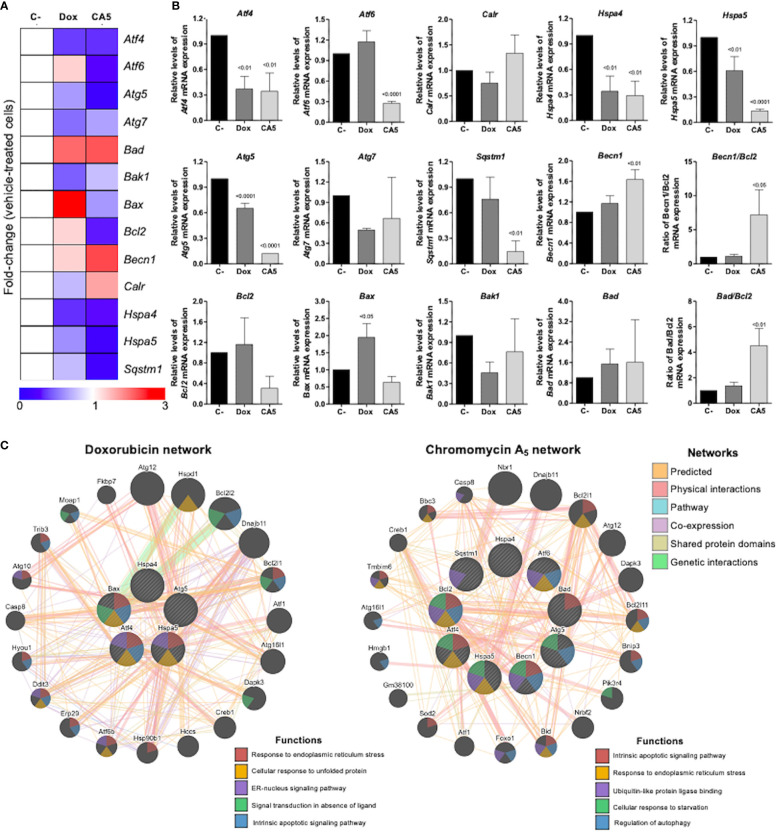
Chromomycin A_5_ modulates transcription genes related to endoplasmic reticulum (ER) stress, autophagy, and cell death. Quantitative RT-PCR was performed for 13 selected genes related to ER stress (*Atf4*, *Atf6*, *Calr*, *Hspa4*, and *Hspa5*); autophagy (*Atg5*, *Atg7*, *Becn1*, and *Sqstm1*); and apoptosis (*Bad*, *Bak1*, *Bax*, and *Bcl2*). **(A)** Heatmap illustrating all selected genes in B16-F10 upon a vehicle, Dox (0.6 μM) or CA5 (0.1 μM) exposure for 24 h. The data are presented as fold-change of the vehicle-treated cells. Downregulated and upregulated genes are illustrated in blue and red, respectively. **(B)** The comparison of selected genes are presented in bar graphs and the *p* values are indicated. **(C)** Network analysis for genes modulated significantly by Dox or CA5 constructed using the GeneMANIA database (https://genemania.org/). The upregulated and downregulated genes in the quantitative RT-PCR are illustrated as strikethrough circles, and the interacting genes included by the software modeling are indicated by strikethrough strands. The main interactions between genes are indicated by colored lines and the five main cellular processes are described in the Figure.

To obtain new insights into the molecular mechanisms involved in the response of B16-F10 cells to CA_5_, we investigated the expression of 13 genes related to autophagy, apoptosis, and ER stress by quantitative RT-PCR. A total of 7 of out 13 genes was significantly modulated by CA5 treatment (6 downregulated [*Atf4, Atf6, Hspa4, Hspa5, Atg5*, and *Sqstm1*] and 1 upregulated [*Benc1*], all p < 0.05), while 5 genes were significantly modulated by Dox treatment (4 downregulated [*Atf4, Hspa4, Hspa5*, and *Atg5*] and 1 upregulated [*Bax*] all p < 0.05) in B16-F10 cells. Of note, treatment with CA5, but nor Dox, significantly increased the *Becn1/Bcl2* and *Bad/Bcl2* ratios (**Figures 5A, B**). The network analysis indicates that CA5 induces more complex relationships, which effectively interconnects the processes of apoptosis, autophagy, and ER stress (**Figure 5C**).

### 3.6 Activation of antigen-presenting cells

**Figure 6:**
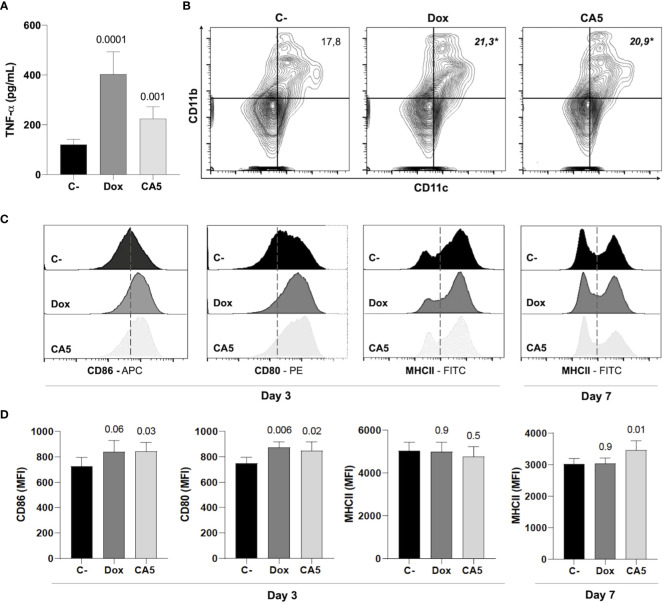
B16-F10 cells exposed to chromomycin A5 induce activation of dendritic cells (DCs). Cells pre-incubated for 24 h with 0.1 μM CA_5_ (CA5) and 0.6 μM doxorubicin (Dox) were added to naïve splenocytes *in vitro* **(A)**, or injected subcutaneously in the right axilla of mice **(B-D)**. **(A)**, Column graph showing TNF-α production by splenocytes of naïve mice incubated with B16-F10 treated cells as determined by ELISA. Cells pre-incubated with DMSO were used as a negative control (C-). **(B)**, representative flow cytometry contour plot graphs of CD11b and CD11c dendritic cells with their mean values in percentage. **(C, D)**, representative flow cytometry histograms of surface markers CD80, CD86 and MHC II and column graphs of their mean fluorescence intensity (MFI) respectively acquired on CD11b^+^CD11c^+^ region. Sterile saline solution injected in right axilla was used as negative control (C-) in **(B-D)**. **(A-C)** data were obtained after incubation 3 days with treated-cells. *p* values of treated groups compared to C- are written above columns of the treated groups. *p < 0.05 in **(B)** N= 5 animals per group.

**Figure 7:**
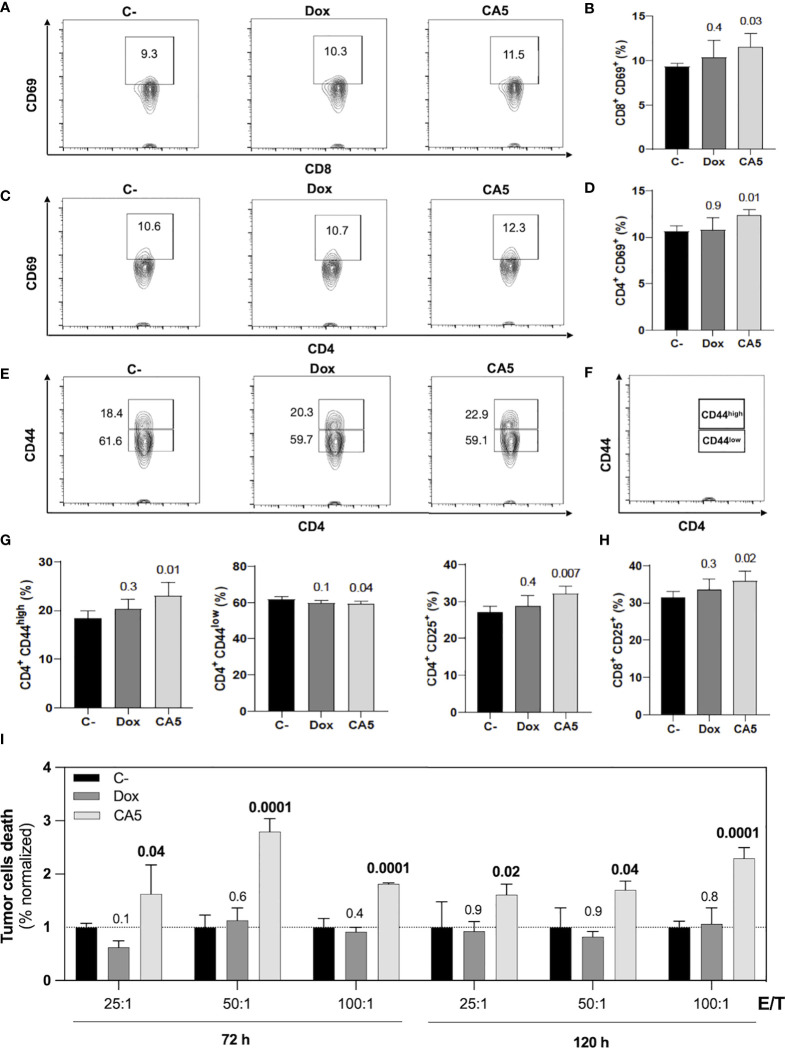
B16-F10 cells exposed to chromomycin A5 induce activation of CD4^+^ and CD8^+^ T lymphocytes and generate cell death in viable B16-F10 cells. Cells pre-incubated for 24 h with 0.1 μM CA_5_ (CA5) and 0.6 μM doxorubicin (Dox), or sterile saline (C-) were injected subcutaneously in the right axilla of mice and their splenocytes were evaluated 7 days post-vaccination. **(A)** Representative contour plot graphs of CD69 surface marker on CD8 T lymphocytes. **(B)** Column graphs showing the percentage of CD8^+^CD69^+^ T lymphocytes. **(C)** Representative contour plot graphs of the CD69 surface marker on CD4 T lymphocytes. **(D)** Column graphs showing the percentage of CD4^+^CD69^+^ T lymphocytes. **(E)** Representative contour plot graphs of CD44 surface marker on CD4 T lymphocytes showing CD44^high^ and CD44^low^ populations and **(F)** illustration identifying CD44^high^ and CD44^low^ populations. **(G)** Column graphs showing the percentage of CD4^+^CD44^high^ T lymphocytes, CD4^+^CD44^low^ T and CD4^+^CD25^+^ T lymphocytes and **(H)** column graphs showing the percentage of CD8^+^CD25^+^ T lymphocytes. **(I)** Column graphs showing cell death of B16-F10 cells after 5h incubation with splenocytes ratios of effector/target (E/T). The splenocytes were previously restimulated with sterile saline (C-), CA5 and Dox for 72h and 120h before the cytotoxic assay. Differences between groups are expressed as *p* values indicated above the compared groups. N= 5 animals per group.

Next, the immune activation by B16-F10 dying cells exposed to CA_5_ was investigated. Splenocytes from naïve mice released TNF-α when incubated with Dox-treated cells ou CA5-treated cells (**Figure 6A**). This result suggests activation of antigen-presenting cells, and it was confirmed by a vaccination assay. Thus, we perform a vaccination assay to verify whether CA5-treated B16-F10 promotes dendritic cell activation *in vivo*. The vaccination of mice with B16-F10 dying cells exposed to Dox and CA_5_ increased the frequency of CD11b^+^CD11c^+^ DC population (**Figure 6B**) and expression of the activation markers CD80 and CD86 on the surface of these cells (**Figures 6B–D**). CD11b^+^/CD11c^+^ DCs of CA_5_ group increased MCH II expression (**Figure 7D**), however Dox group did change it comparing to saline group. Additionally, vaccination with B16-F10 dying cells exposed to CA_5_ also activated CD11b^-^CD11c^+^ population (**Figure 6B**) by increasing expression of CD80 and CD86 3 days post vaccination (data not shown), which was not observed to Dox group. These data suggest CA5-treated melanoma cells modulate innate antitumor immunity activating antigen presenting cells.

### 3.7 Vaccination of mice with B16-F10 dying cells exposed to CA_5_ induces T cell activation and protection against tumor challenge with viable B16-F10 cells

Mice vaccinated with B16-F10 dying cells exposed to CA_5_ increased activation markers on splenic CD4^+^ and CD8^+^ T cells in comparison with the saline group (**Figures 7A–H**). The Dox group did not show difference to the saline group. In the CA5 group, CD4^+^ and CD8^+^ T cells augmented expression of CD69 (**Figures 7A–D**) and CD25 (**Figure 7H**). CD69 is an early lymphocyte activation marker due to its rapid appearance on the surface of the plasma membrane after stimulation. CD69 is necessary for the traffic of CD4^+^ effector T cells to the bone marrow, mainly for the relocation and persistence of their interaction with stromal cells, such as memory helper T cells (42). Recent antigenic stimulation could increase CD25^+^ on T cells and it also has functional significance in regulating T cell proliferation (43). CD4^+^ T cells also increased CD44^high^ followed by a decrease of CD44^low^ subpopulations (**Figures 7E–G**) on CA_5_ group. The CD8^+^ did not change the CD44 expression on Dox or CA5 (data not shown). CD44 is found in naïve cells and in activated cells, with the naïve population being characterized as CD44^low^ and the activated one as CD44^high^. High expression of CD44 is considered a reliable identification for memory T cells in mice, both CD4^+^ and CD8^+^ (44). Then, these results indicate that vaccination with CA5-treated melanoma cells also promotes adaptive immune response by broad activation of T cells.

**Figure 8:**
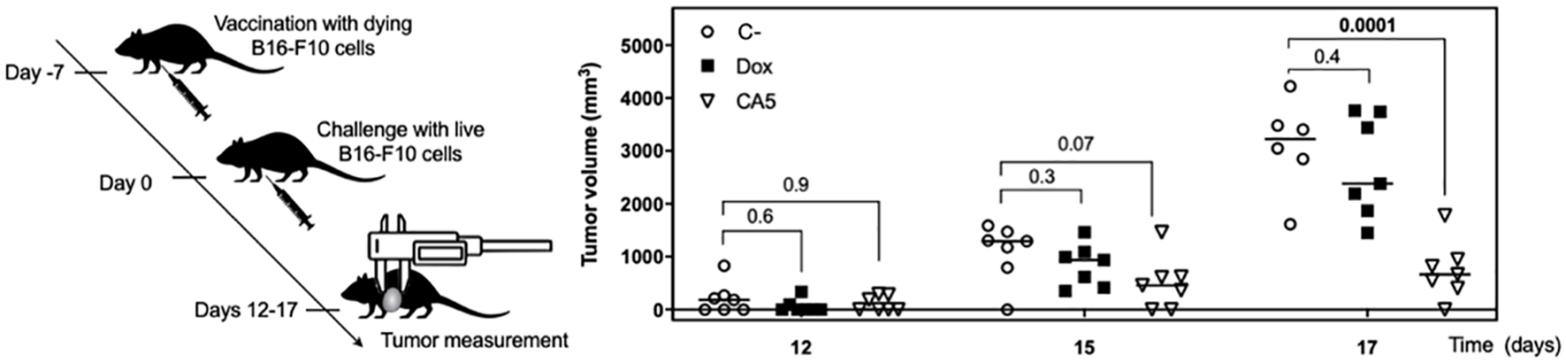
Vaccination with dying B16-F10 cells exposed to chromomycin A_5_ confers mice resistance against live B16-F10 cells. At day -7, 1.8 × 10^5^ cells pre-incubated for 24 h with 0.1 μM CA_5_ (CA5) and 0.6 μM doxorubicin (Dox) were injected subcutaneously in the right axilla of mice. 7 days after vaccination (day 0) mice were challenged with 1 × 10^5^ live B16-F10 cells in the left axilla. The mice of the negative control group (C-) were injected with saline solution at -7 day and live B16-F10 cells at day 0. N= 7 mice/group. Tumor growth was monitored until day 17. Differences between groups are expressed as *p* values indicated above the compared groups.

The cytotoxic activity of splenocytes from vaccinated mice was assessed against tumor cells *in vitro*. In accordance to lymphocyte activation profile, the splenocytes of CA_5_ group killed B16-F10 cells, while Dox group did not (**Figure 7I**). Therefore, we confirmed the antitumor immune response of mice vaccinated with B16-F10 exposed to CA_5_. The vaccination assay using cells dying triggered by a cytotoxic agent followed by challenge with the viable cells of the same tumor cell line is the gold standard technique for ICD confirmation (7). CA_5_-exposed cells injected 7 days before the challenge with B16-F10 viable cells developed a significant tumor growth control protection (**Figure 8**). This vaccination effect was not observed with mice of the Dox group.

## 4 Discussion

AMM lacks a therapeutic option to convert immune-cold into immune-hot tumors to improve the clinical response of the patients who do not respond to the current arsenal available including immunotherapy (45). The identification of ICD inducers could fill this need by combining two useful effects at once, direct cytotoxicity against tumor cells and release of immunological activating signals. This type of regulated cell death allows the proper activation of the immune system, which in your turn, eliminates tumor cells resistant to chemotherapy. This mechanism is related to more effective and long-lasting responses (46). Herein we investigated the ICD induction of four chromomycins obtained from the marine bacterium *Streptomyces* sp. BRA-384 against metastatic melanoma.

Initially, the cytostatic and cytotoxic profiles of CA_5-8_ were investigated with increasing time exposure, and it was observed a time-dependent effect in the nM range. Notably, CA_5_ and CA_7_ depicted cytotoxicity in low time-exposure of 4 h and 8 h respectively (**Figure 1A**). Additionally, CA_5-8_ completely inhibited colony formation of tumor cells after 24 h incubation (**Figure 1B**). These data highlighted a favorable cytotoxic feature of chromomycins as anticancer compounds, which must achieve therapeutic plasma levels in a short time window due to toxicity.

The most reliable approach to the initial identification of ICD still consists in performing multiple phenotypic assays to evaluate autophagy, apoptosis, and releasing of DAMPs on treated cells *in vitro* (12). In general, cells treated with CA_5-8_ and Dox depicted some ICD features such as regulated cell death (**Figure 2**), cell stress related to autophagy (**Figure 3**) and externalization of CRT, and releasing of HMGB1 (**Figures 4B, F**). Notably, CA_5_-treated cells depicted the most consistent ICD profile, filling all phenotypic features investigated so far, followed by CA_6_ and CA_7_, CA_8_ and Dox (**Figure 4G**). Dox failed to activate caspase 3 or to increase cleaved PARP1 on B16-F10 cells, important apoptosis markers (47, 48) and Dox and CA_6-8_ all failed to release ATP, an essential DAMP involved in the ICD (7, 49, 50). CA_5_ induced ATP release, ERp57 externalization, and phosphorylation of eIF2α (**Figures 4C, I, M** respectively). Dox increased eIF2α levels, without significant phosphorylation at serine 51 (**Figures 4K, M**). Analyses of anticancer ICD inducers revealed eIF2α phosphorylation mediated by eIF2α kinase-3 (EIF2AK3), but no other signs of ER stress are related to CRT exposure (40, 41, 51). Furthermore, machine-learning approaches revealed eIF2α phosphorylation as the sole ER stress response relevant to the algorithm with downstream consequences including CRT exposure, stress granule formation, and autophagy induction (40, 41). Although Dox did not induce a significant increase of phosphorylation of eIF2α, it induced CRT externalization (**Figure 4F**) and increased the high granularity population (**Figures 3A, B**) and autophagy (**Figures 3C, D**). This conflicting data could be explained as a masking effect of the increase eIF2α in Dox-treated cells (**Figure 4K**), which could produce biologically relevant phosphorylation of eIF2α as detected by increased ecto-CRT and autophagy.

CA_5_ and doxorubicin altered expression of transcription of 13 selected genes related to autophagy, ER stress, and apoptosis. However, CA_5_-treated cells changed most of the genes evaluated and increased the Becn1/Bcl2 and Bad/Bcl2 ratios (**Figures 5A, B**) and depicted a wider interconnected network among apoptosis, autophagy, and ER stress than doxorubicin-treated cells (**Figure 5C**). It is worth highlighting the cellular response to starvation along with ER stress and apoptosis, as a putative indication of a more intense stress response on CA_5_-treated cells in comparison to Dox. Activating transcription factors *Atf4* and *Atf6* were downregulated on CA5 cells (**Figure 5B**). Dox treatment also decreased *Atf4*, however did not alter *Atf6*. ICD inducers, such as anthracyclines, enhance phosphorylation of eIF2α, but fail to stimulate other ER stress signs including the transcriptional activation of activating transcription factor 4 (ATF4) and the proteolytic cleavage of activating transcription factor 6 (ATF6) (40, 41).

**Figure 9:**
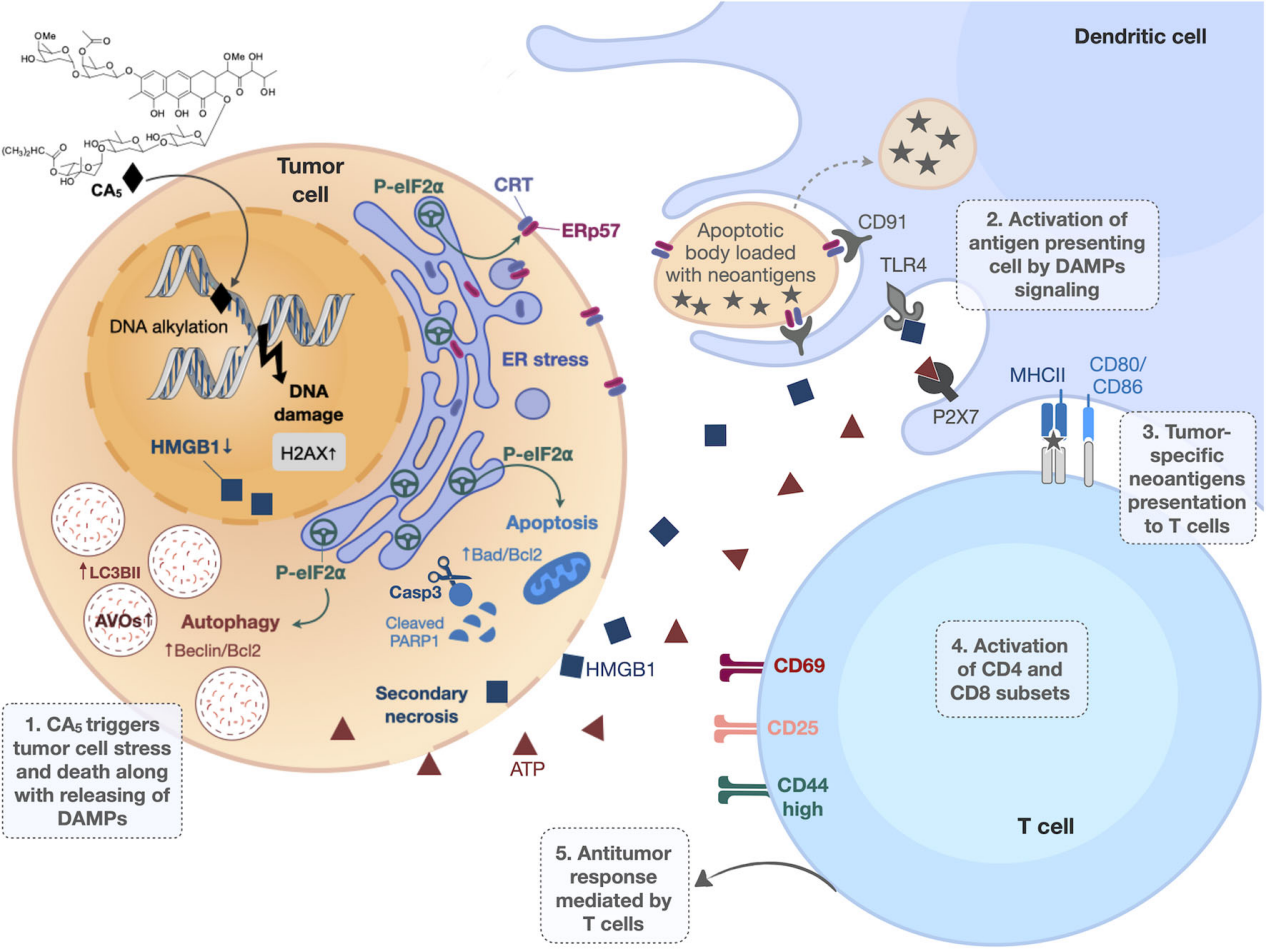
Overview of immunogenic cell death (ICD) induced by chromomycin A_5_ (CA_5_) on melanoma. B16-F10 cells treated with CA_5_ displayed cell stress and cell death along with release of damage-associated molecular patterns (DAMPs). The phosphorylation of eIF2α, due to endoplasmic reticulum (ER) stress, drives crucial immunogenic events, as externalization of the “eat me” signals calreticulin (CRT) and ERp57, autophagy and apoptosis. Dendritic cells are activated by DAMPs and present tumor antigens to T lymphocytes *via* MCH in the presence of co-stimulatory molecules (CD80 or CD86). The activation of T lymphocytes by dendritic cells confers antitumor response. AVOs, acidic vesicular organelles; Casp3, caspase 3; PRRs, pattern recognition receptors, HMGB1, high mobility group box 1; ATP, adenosine triphosphate.

Further investigation confirmed a proper activation of the immune system by B16-F10 cells exposed to CA_5_. First, it was observed the releasing of TNF-α by splenocytes cultured in the presence of B16-F10 pre-exposed to CA_5_ and Dox, which suggested the activation of dendritic cells. Dying cells undergoing bona fide ICD must effectively recruit and activate both APCs and lymphocytes without any external adjuvants. The vaccination assay is the gold standard method to confirm ICD, due to its complex spatio-temporal nature (5, 7, 49). Then multiple vaccination assays were conducted with the administration of B16-F10 pre-exposed to CA_5_ or Dox on mice, followed by the evaluation of splenocytes after different time points. CA_5_ induced the increase and activation of conventional type 2 DCs (cDC2), CD11b^+^CD11c^+^ (**Figure 7**). cDC1 (CD11b^-^CD11c^+^) were also activated in CA5 group without a quantitative change in this population. cDCs present tumor antigens and secrete cytokines that regulate T cell survival and activation. cDC1 are important for antitumor immunity and are associated with increased overall survival of oncologic patients (52). The antigen presentation depends on the tumor neoantigens loaded on MHC II along with another costimulatory molecule, such as CD80 or CD86 (8, 53). CA_5_ group showed elevated expression of these activation markers on DCs. The Dox group increased cDC2 subpopulation and increasing of CD80 and CD86, however, it did not change the MCH II expression. Increased T lymphocytes activation was also observed seven days after vaccination on CD4^+^ and CD8^+^ T cells of the CA5 group, while Dox failed to increase the early activation markers CD69, CD25 or CD44 (**Figure 8**) at this moment. Despite T cells activation of CA5 group, their effector function still need to be confirmed. Then the antitumor immune response of vaccinated mice with CA5-treated cells was evaluated. In accordance with the lymphocyte activation profile, the splenocytes of CA5 killed B16-F10 cells *in vitro*, while Dox group did not (**Figure 7I**). A summary of cellular changes induced by CA_5_ on B16-F10 cell line and immune system activation events investigated is illustrated in the **Figure 9**. The vaccination efficacy was also evaluated by challenging vaccinated mice inoculating live cells of the same lineage. C57BL/6 mice vaccinated with CA_5_-treated cells controlled tumor growth efficiently (**Figure 8**). At day 17 the mean tumor volume of mice of the CA5 group was significantly lower (*p* = 0.0001) than the mean tumor volume of the C- group. Actually, animals from the CA5 group showed only 20% of the mean saline tumor volume, and one animal did not develop a tumor at all. This result confirms CA_5_ as a bona fide ICD inducer. Tumors of the Dox group did not show a significant difference from the negative control (p = 0.4). Gomez-Cadena et al. (28) reported significant tumor control of C57L/6 mice vaccinated with Dox-treated B16-F10 cells. In their study, the cell treatment with doxorubicin was longer (48 h), and caspase 3 activation confirmed apoptosis induction, which was not observed herein. However, the ATP levels in supernatants of Dox-treated cells did not increase either, similar to our results as we found in the present study.

Fine-tuning *in vitro* conditions to confirm the induction of ICD is challenging and some studies could fail to demonstrate it depending on the histological origin of cells and time and concentrations of exposure used as well (54). Most ICD inducers, such as doxorubicin, oxaliplatin, bortezomib and vinca alkaloids, were identified using tumor cell lines from different tumor origins of clinical practice (54, 55). Although this approach generated robust knowledge about ICD inducers initially, it led to delayed identification of some important ICD inducer anticancer agents, including paclitaxel and cisplatin (56, 57). Similarly, other chromomycins, including CA_7-8_ studied here, also induce ICD depending on experimental design; however further studies are needed to fully characterize ICD triggered by chromomycins. Additionally, the suboptimal results we obtained with melanoma cells exposed to doxorubicin, an important ICD inducer used in the treatment of several solid and hematological cancers (e.g. breast, ovary, prostate and multiple myeloma), also illustrates the challenge of identifying experimental conditions that trigger ICD.

A few chemotherapeutic agents are known to induce ICD, and they demonstrate remarkable clinical performance (10), CA_5_ shows evidence of ICD and thus is a highly promising candidate for AMM and deserves further preclinical studies. It is also worth highlighting the supply as one important bottleneck to the preclinical and clinical development of pharmaceuticals (58). We obtained CA_5_ for this study using a sustainable and easily scalable technique (23), and its supply for studies *in vivo* is quite feasible. In summary, we identified CA_5_ as a bona fide inducer of ICD in metastatic melanoma model. Further *in vivo* studies with CA_5_ are necessary to evaluate antitumor activity, toxicity, and survival, as well as the effect of CA_5_ associated with immunotherapy.

## Data availability statement

The raw data supporting the conclusions of this article will be made available by the authors, without undue reservation.

## Ethics statement

The animal study was reviewed and approved by Animal Ethics Committee of the Federal University of Ceara (No 3000310818) and Animal Welfare Committee of the Ribeirão Preto Medical School, University of São Paulo (No 226/2018).

## Author contributions

KGF: Methodology, Validation, Writing - Original Draft, Formal analysis, Investigation, Visualization. EE: Methodology, Validation, Investigation, Formal analysis. KSF: Methodology, Validation, Investigation, Formal analysis, Writing. JL: Methodology, Validation, Investigation, Formal analysis. FP: Methodology, Validation, Investigation. OP: Validation, review the manuscript. FC: Experimental design, Validation, Review the manuscript, Funding acquisition. JM-N: Methodology, Validation, Formal analysis, Investigation. DW: Conceptualization, Funding acquisition, Supervision, Investigation, Validation, Writing - Review & Editing. All authors contributed to the article and approved the submitted version.

## Funding

This study was financed in part by the Coordenação de Aperfeiçoamento de Pessoal de Nível Superior - Brasil (CAPES) - Finance Code 001, Instituto Nacional de Ciência e Tecnologia (INCT BioNat-CNPq/FAPESP, No. 465637/2014-0) and Fundação de Amparo à Pequisa do Estado de São Paulo (2019/23864-7). This work was also funded by FAPESP no. 2013/08216-2 (Center for Research in Inflammatory Diseases).

## Acknowledgments

We thank to Dr. Margo Haygood for reviewing the manuscript. The authors also thank the Multi-User Facility of Drug Research and Development Center of Federal University of Ceará for technical support.

## Conflict of interest

The authors declare that the research was conducted in the absence of any commercial or financial relationships that could be construed as a potential conflict of interest.

## Publisher’s note

All claims expressed in this article are solely those of the authors and do not necessarily represent those of their affiliated organizations, or those of the publisher, the editors and the reviewers. Any product that may be evaluated in this article, or claim that may be made by its manufacturer, is not guaranteed or endorsed by the publisher.

## Supplementary material

The Supplementary Material for this article can be found online at: https://www.frontiersin.org/articles/10.3389/fimmu.2022.941757/full#supplementary-material

## Abbreviations

AMM, advanced metastatic melanoma; AO, acridine orange; APC, antigen presenting cell; AVOs, acidic vesicular organelles, CRT, calreticulin; DAMPs, damage-associated molecular patterns; eIF2α, eukaryotic initiation factor 2α; ER, endoplasmic reticulum; HMGB1, high mobility group box-1; ICD, immunogenic cell death; PD-1, programmed death-1; PD-L1, programmed death-1 ligand.
