# Supplementary figures and images for: Chromomycin A5 induces bona fide immunogenic cell death in melanoma
Source: Front Immunol. 2022 Nov 9;13:941757. doi: 10.3389/fimmu.2022.941757 (PMC9682167; doi:10.3389/fimmu.2022.941757)

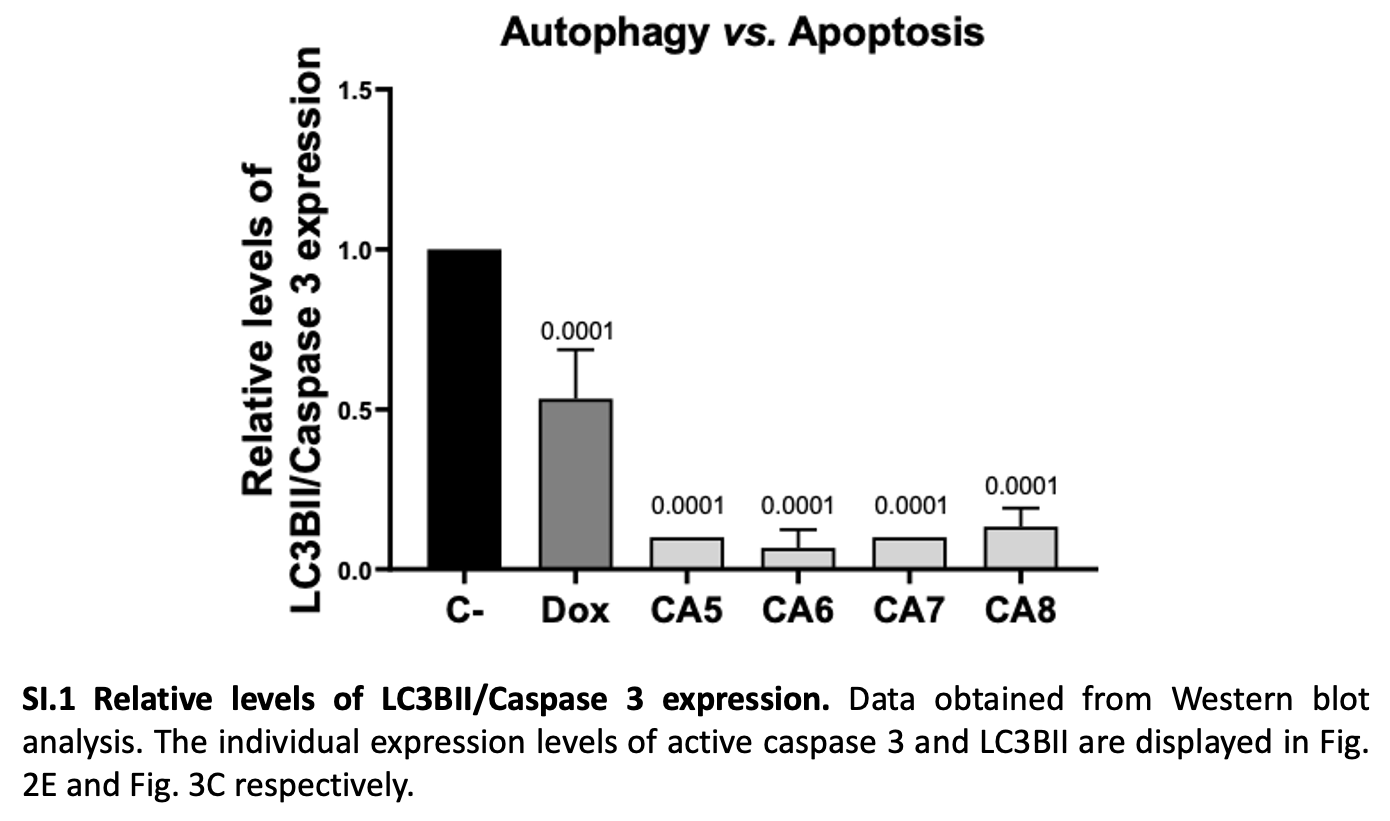

Supplement: Supplementary file 1 [file Image_1.png]

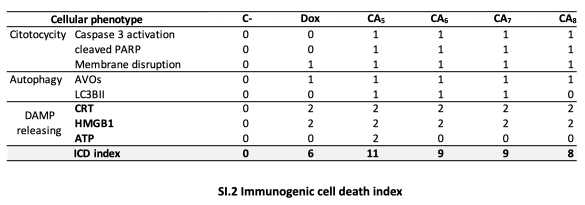

Supplement: Supplementary file 2 [file Image_2.tiff]
